# Mach Fronts in Random Media with Fractal and Hurst Effects

**DOI:** 10.3390/fractalfract5040229

**Published:** 2021-11-18

**Authors:** Junren Ran, Martin Ostoja-Starzewski, Yuriy Povstenko

**Affiliations:** 1 Department of Mechanical Science & Engineering, University of Illinois at Urbana-Champaign, Urbana, IL 61801, USA; 2 Department of Mechanical Science & Engineering, Institute for Condensed Matter Theory and Beckman Institute, University of Illinois at Urbana-Champaign, Urbana, IL 61801, USA; 3 Faculty of Science and Technology, Jan Dlugosz University in Czestochowa, 42-200 Czestochowa, Poland

**Keywords:** Mach front, supersonic, random media, fractal, Hurst effect

## Abstract

An investigation of transient second sound phenomena due to moving heat sources on planar random media is conducted. The spatial material randomness of the relaxation time is modeled by Cauchy or Dagum random fields allowing for decoupling of fractal and Hurst effects. The Maxwell–Cattaneo model is solved by a second-order central differencing. The resulting stochastic fluctuations of Mach wedges are examined and compared to unperturbed Mach wedges resulting from the heat source traveling in a homogeneous domain. All the examined cases are illustrated by simulation movies linked to this paper.

## 1. Introduction

Continuum mechanics of homogeneous media has been the basis of studies of shock formation due to sources moving faster than the speed of sound. Such studies belong to aerodynamics. However, if a generalization to heterogeneous media is considered, the subject of such shocks in random media would have its basis in a general field of *waves in random media* (or *stochastic wave propagation*). While various investigations have been carried out in this area, to the best of our knowledge, no studies of shock and Mach fronts due to sources moving in random media have ever been reported. In particular, random media with fractal (i.e., hierarchical-type multiscale) and Hurst (i.e., long-range memory) effects offer an additional difficulty.

The basic motivation for studying waves in such media comes from the fact that such geometric characteristics appear in nature, both in biological and geologic structures, as well as atmospheric turbulence [1–4]. In addition, ref. [4] reported that heat conduction in a porcine muscle follows a damped wave equation of *second sound,* and this finding is consistent with other studies, e.g., [5,6]. At present, it is not known whether this equation would also correctly model the heat transfer in electrosurgery, where a surgeon may cut a living tissue at several centimeters per second. However, given the observation that signals in soft tissues may travel slower than a surgeon’s hand, there may occur supersonic (or even hypersonic) heat propagation phenomena. Thus, the present paper is a stepping-stone in that direction.

Overall, this paper is organized as follows. Section 2 gives the problem formulation in a stochastic framework. Section 3 introduces the random field (RF) models of Cauchy and Dagum types. Section 4 outlines the numerical treatment of the telegraph equation. Section 5 gives the numerical results in terms of the animations of temperature fields and provides comparisons between such fields and the reference solution on a homogeneous medium. Finally, Section 6 concludes this paper.

## 2. Problem Formulation

**Figure 1.**
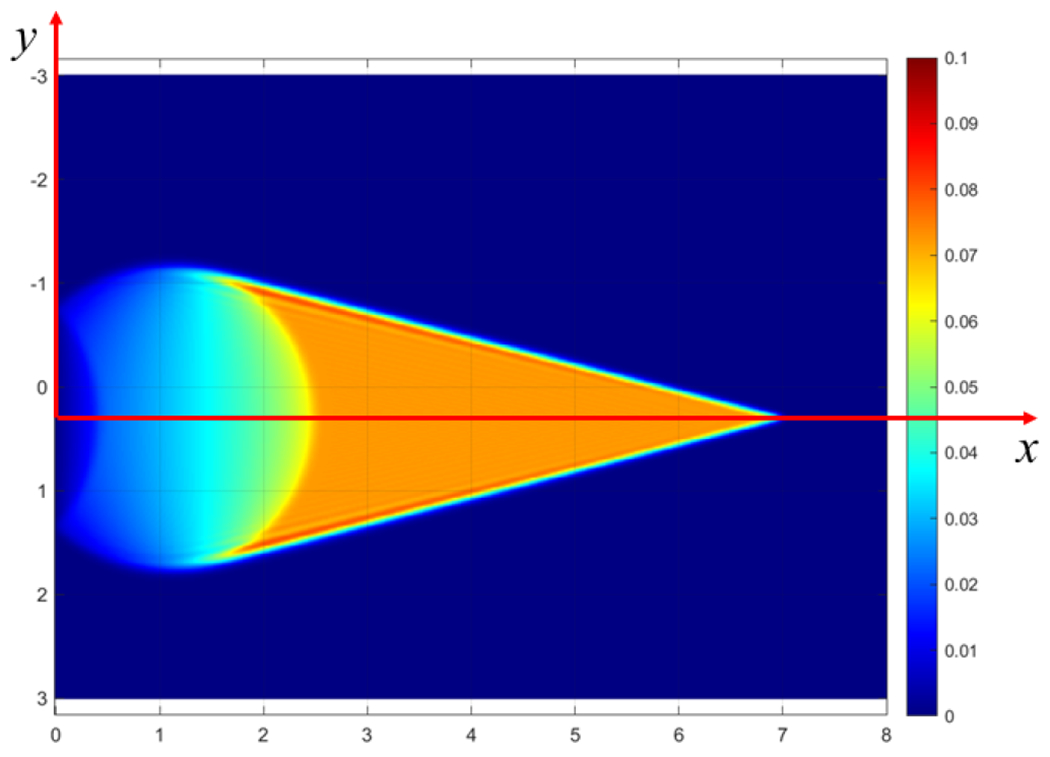
Simulation result at *t* = 3 in a homogeneous medium of *τ* = 4.

The investigation concerns second sound phenomena in two dimensions (2d), i.e., those governed by a telegraph partial differential equation for the temperature field *T* in a planar body domain *B* (Figure 1).

(1) $$\frac{\partial T}{\partial t}+\tau \frac{\partial^{2}T}{\partial t^{2}}=\kappa \nabla^{2}T+Q(x,t)\;\mathrm{in}\;B\subset {\mathbb{R}}^{2}\;T=0\;\mathrm{on}\;\partial B$$

where ∇^2^ is the Laplacian in the (*x, y*)-plane, *Q* is the heat source, *κ* the thermal diffusivity is taken = 1, while *τ*, the relaxation time, is taken as a random field (RF). The second sound is a thermal wave resulting from the hypothesis that the temperature gradient is proportional not only to the heat flux (as in Fourier’s law) but also to the rate of heat flux multiplied by a relaxation time. As a result, the heat conduction is governed by a hyperbolic rather than a parabolic partial differential equation. The relaxation times are on the order of seconds and even tens of seconds in polymers [4–6]. The hyperbolic transfer equation was first obtained by Fock [7], followed by Davydov [8], Cattaneo [9,10] and Vernotte [11].

The RF *τ* is taken according to a Reynolds decomposition:

(2) $$\tau =\langle \tau \rangle +\tau^{\prime },\;\langle \tau^{\prime }\rangle =0.$$

In general, $\tau^{\prime }=\left\{\tau^{\prime }(x,\omega );\omega \in \text{Ω}\right\}$ is an RF of position x(∈B), where *ω* is a realization drawn from a probability space (Ω,F,P).

We consider situations where *Q* moves faster than the speed of second sound *c*, implying the supersonic effects. Given that $c=\sqrt{\kappa /\tau }$, *c* is a random field, so these effects are stochastic in space–time. Hence, the basic question is: *How are the Mach wedges affected by the medium having a random multiscale structure with long-range memory?* Mathematically, the RF *τ* is generated by employing Cauchy or Dagum correlation functions, where the fractal dimension *D* and the Hurst parameter *H* can be varied independently, i.e., without imposing the self-affine geometry [12,13]. Note that the conventional random process/field models such as white-noise or Ornstein–Uhlenbeck, while very common in the literature, do not exhibit these interesting effects.

From a general perspective, the present study continues our investigations of (i) structural mechanics problems, (ii) dynamical systems, (iii) waves and wavefronts and (iv) fracture on tensor-valued random fields (see, e.g., [14]). Only in the simplest cases of (i) and (ii) are explicit analytical solutions possible, whereby we note that even the explicit spectra of Cauchy and Dagum models are formidable mathematical challenges. Therefore, we employ a computational approach to simulate realizations of RFs and then to solve for wave fields due to sources moving on such fields.

Generally, the governing field equation of deterministic continuum physics in a homogeneous medium can be written as

(3) ℒT=Q,

where *ℒ* is a partial differential operator governing the second sound phenomenon; recall (1). If we introduce spatial randomness into the material properties appearing in the partial differential operator, then (3) is replaced by a stochastic field equation incorporating the spatial fluctuation

(4) ℒ(ω)T=Q;ω∈Ω.

Here, *ω* is a single realization of the sample space Ω which together with a *σ*-algebra *S* and a probability measure *P* on it forms a probability space (Ω,F,P). At this stage, a Gaussian measure will be assumed, although, if more information about the material randomness is available, other types of probability distribution are possible. The space (Ω,F,P) defines a random medium ℬ={B(ω);ω∈Ω}. The ensemble average (i.e., the stochastic expectation) of ℒ is

(5) $$\langle ℒ\rangle =\int_{\text{Ω}}ℒ(\omega )dP,$$

and, as always for stochastic systems, one is first interested in the average displacement response 〈T〉 before inquiring about the second and higher moments, and, ideally, about the probability distribution of the response. The above straightforward averaging of (4) leads to a deterministic field equation

(6) $$\langle ℒ\rangle T_{\text{det}}=f,$$

where *T*_det_ is the deterministic (and supposedly right…) solution. However, the correct average solution, 〈T〉, of the stochastic problem (4) is formally obtained by first inverting (4), then averaging, and then inverting again:

(7) $$\langle {ℒ}^{-1}\rangle {}^{-1}\langle T\rangle =f.$$

In general, there is no closed-form solution to (7). Therefore, the basic question of stochastic field theories is: If one replaces $\langle {ℒ}^{-1}\rangle {}^{-1}\;\text{by}\;\langle ℒ\rangle$, how different will the solutions 〈T〉 and $T_{\text{det}}$ be?

In the following, we focus on a related issue from a geometric standpoint: *To determine the effect of random multiscale structure with long-range memory on the Mach wedges.* This will be done by comparing the realizations of temperature RFs T={T(x,t,ω);ω∈Ω} in the random medium setting with the reference solution in the homogeneous reference medium defined by τ=〈τ〉. Effectively, since analytical solutions are impossible, the study method is of the Monte Carlo type. Note that the analytical solution elucidating the Doppler effect in the subsonic range of the second sound in a homogeneous 1d medium has recently been obtained [15].

## 3. Random Fields

**Figure 2.**
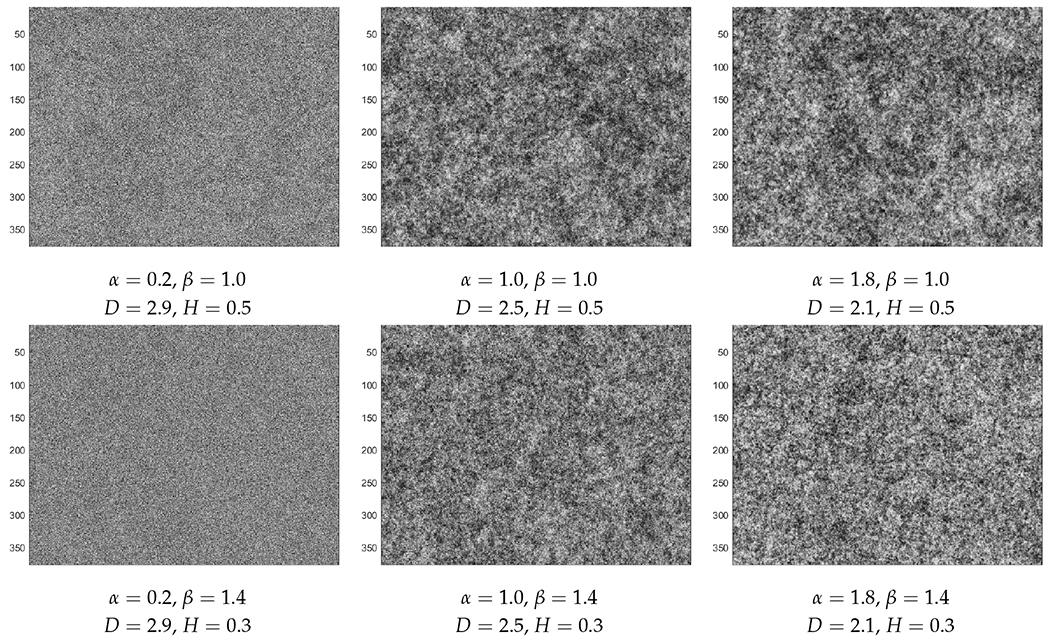

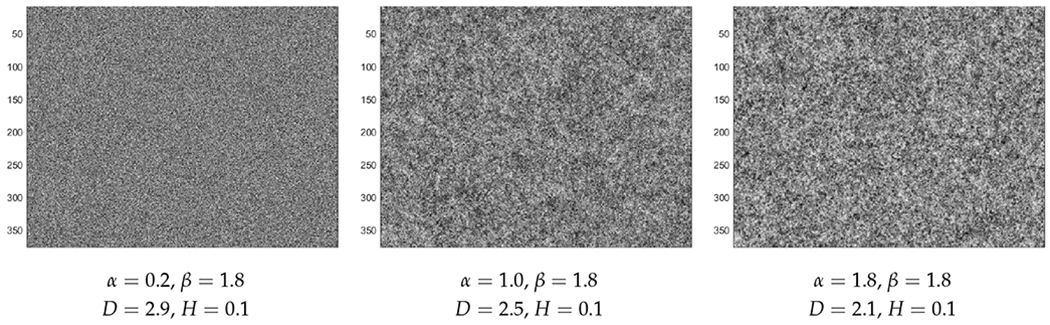
Realizations of Cauchy random fields with columns (from left to right) at *α* = 0.2, 1.0, 1.8 and horizontal rows (from top to bottom) *β* = 1.0, 1.4, 1.8.

**Figure 3.**
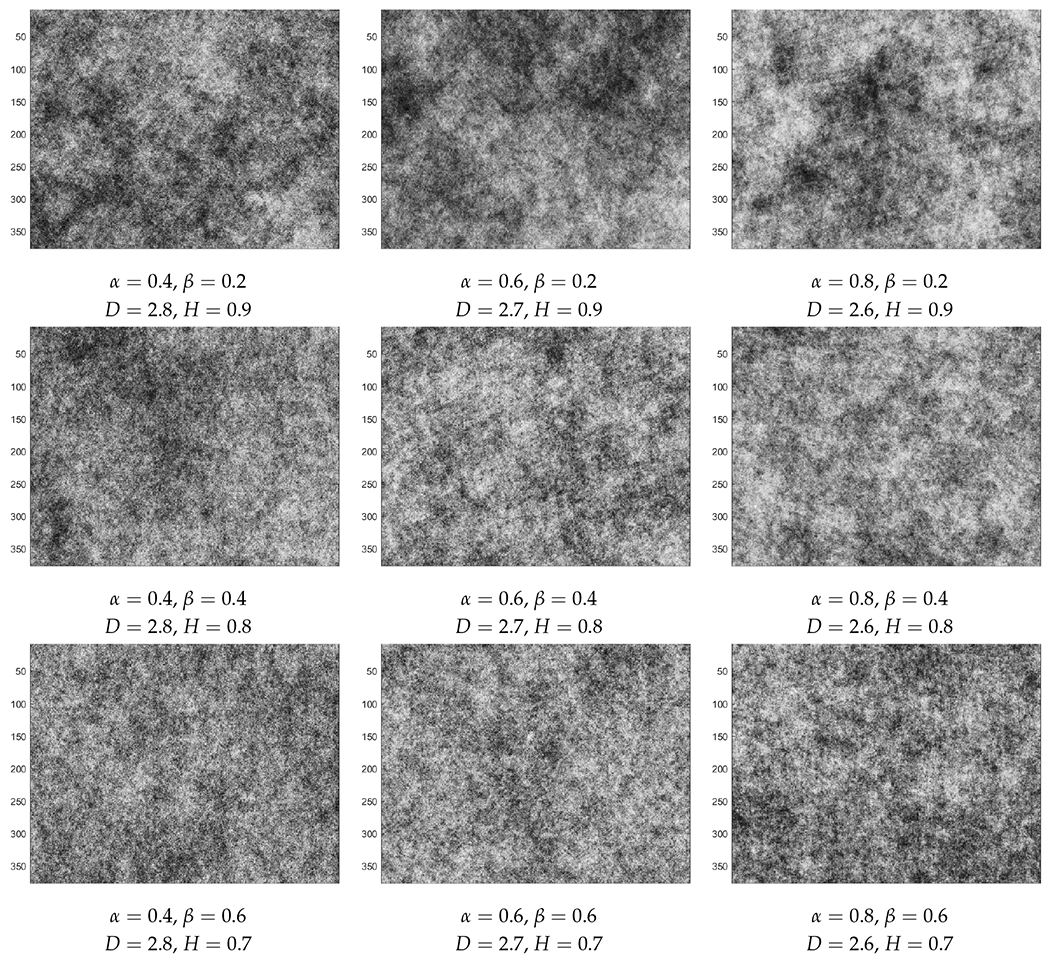

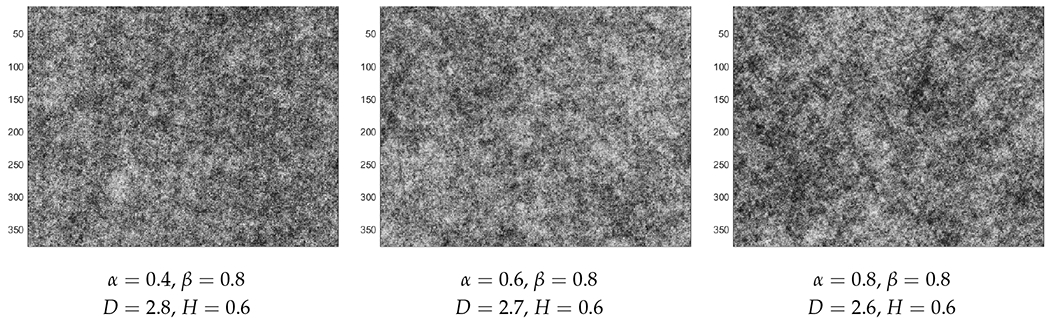
Realizations of Dagum random fields with columns (from left to right) at *α* = 0.4, 0.6, 0.8 and horizontal rows (from top to bottom) *β* = 0.2, 0.4, 0.6, 0.8.

Since many conspicuous patterns in nature can be described by fractal geometry and Hurst characteristics, two RF models which can capture such effects are considered here: Cauchy and Dagum [12,13]. Realizations of these RFs for a wide range of fractal dimension *D* and Hurst parameter *H* are displayed in Figures 2 and 3. The fractal dimension denoted by *D* is a roughness measure with *D* ∈ [2, 3) for two-dimensional systems. A larger *D* indicates a rougher surface. The Hurst parameter denoted by *H* describes the long-range dependence. In general, an anti-persistent system is associated with *H* ∈ (0, 0.5), but a persistent system with *H* ∈ (0.5, 1). For reference, the case of *H* = 0.5 in a 1d RF (i.e., a random process), corresponds to a symmetric random walk without any long-range dependence.

The simplest model one can consider is the white noise RF (i.e., lacking any spatial correlations). Its covariance function is

(8) $$C_{𝒲𝒩}(r)=\delta (r),$$

where *δ* is the Dirac delta function.

The covariance function for a Cauchy RF is

(9) $$C_{𝒞}(r)={\left(1+r^{\alpha }\right)}^{-\frac{\beta }{\alpha }},\;r\geq 0,\alpha \in (0,2],\beta >0.$$

The covariance function for a Dagum RF is

(10) $$C_{𝒟}(r)=1-{\left(1+r^{-\beta }\right)}^{-\frac{\alpha }{\beta }},\;r\geq 0,\alpha \in (0,1),\beta \in (0,1].$$

Both Cauchy and Dagum RF models are capable of decoupling the fractal dimension *D* from the Hurst coefficient *H*; that is, one can independently choose *D* and *H*. With *n* being the dimensionality of the material domain on which the RF is defined (i.e., *n* = 2 for ℝ^2^), the relationships between (*D*, *H*) and (n,α,β) in these two covariance functions are given as

(11) $$D=n+1-\frac{\alpha }{2},\;\text{and}\;H=1-\frac{\beta }{2}.$$

These two equations show that the parameters alpha and beta correspond (and control), respectively, the fractal dimension and the long memory. All the combinations of *α* and *β* for both Cauchy and Dagum RFs are generated using the RF package in R [16]. It is a freeware environment for statistical computing and graphics providing, among others, a convenient tool for simulation of a wide range of random fields.

## 4. Numerical Treatment of Telegraph Equation

The problem to solve is:

(12) $$\frac{\partial T}{\partial t}+\tau \frac{\partial^{2}T}{\partial t^{2}}=\nabla^{2}T+Q\;\mathrm{in}\;B=[0,8]\times [-3,3]\mathrm{cm},\;T=0\;\mathrm{on}\;\partial B,$$

where $Q(x,t)=Q\left(x-vt,y_{0}\right)$ is the heat point source moving at a constant speed *v* = 2 cm/s in the +*x* direction at level *y*_0_ (on the horizontal center line), and *τ* is an RF.

To obtain the numerical solution, we first spatially discretize Equation (12) on a 500 × 375 grid, where Δx=Δy=0.016cm, with the second-order central differencing:

(13) $$\nabla^{2}T_{ij}=T_{i-1,j}-2T_{i,j}+T_{i}$$

This results in a second-order system in the form:

(14) $$\underline{T_{t}}+C\underline{T_{tt}}=A\underline{T}+\underline{Q}$$

where *A* is the Laplacian operator from above, and *C* is the matrix containing material properties *τ* at each grid point.

For the temporal discretization, we first reduce the system to first order in time by constructing a linear system:

(15) $$\underset{M}{\underset{︸}{\left[\begin{array}{cc} I & C\\ I & 0 \end{array}\right]}}\;\underset{{\underline{\tilde{T}}}_{t}}{\underset{︸}{\left[\begin{array}{c} {\underline{T}}_{t}\\ {\underline{T}}_{tt} \end{array}\right]}}=\underset{K}{\underset{︸}{\left[\begin{array}{cc} A & 0\\ 0 & I \end{array}\right]}}\;\underset{\underline{\tilde{T}}}{\underset{︸}{\left[\begin{array}{c} \underline{T}\\ {\underline{T}}_{t} \end{array}\right]}}+\underset{\underline{g}}{\underset{︸}{\left[\begin{array}{c} \underline{Q}\\ 0 \end{array}\right]}}.$$

where the first equation in (15) is equivalent to Equation (14) and the second equation is ${\underline{T}}_{t}={\underline{T}}_{t}$; *I* is the identity matrix. Thus, we are able to time-step the newly constructed vector $\underline{T}$ with its time derivative:

(16) $${\underline{\tilde{T}}}_{t}=M^{-1}(K\underline{\tilde{T}}+\underline{g})\;\mathrm{or}\;\frac{d{\underline{\tilde{T}}}_{t}}{dt}=L\underline{\tilde{T}}+\underline{\tilde{g}}.$$

Upon inspection of the spectrum of *L*, through simple eigen-decompositions, we can see that it has imaginary eigenvalues, which is as expected from the hyperbolic nature of the equation, since the diffusion equation gives negative real eigenvalues and wave equation gives imaginary eigenvalues. Therefore, Runge–Kutta 4 is used as the time-stepper because its stability region covers the imaginary axis, with Δ*t* = 0.008 s where stability can be easily ensured.

## 5. Simulation Results

**Figure 4.**
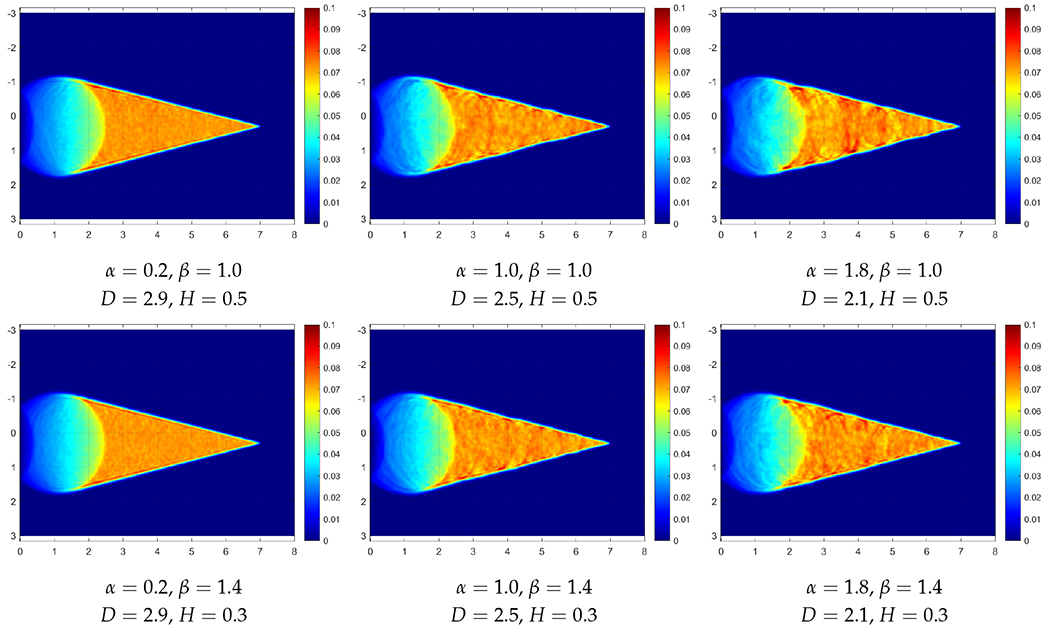

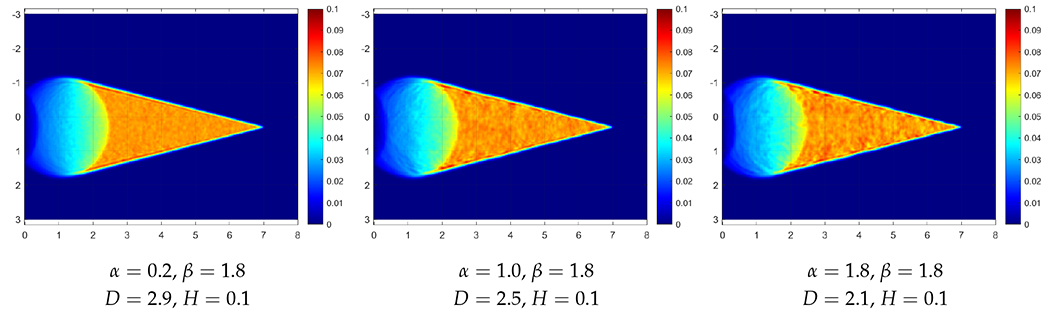
Simulation result at t = 3 in Cauchy random fields with columns (from left to right) at *α* = 0.2, 1.0, 1.8 and horizontal rows (from top to bottom) *β* = 1.0, 1.4, 1.8.

**Figure 5.**
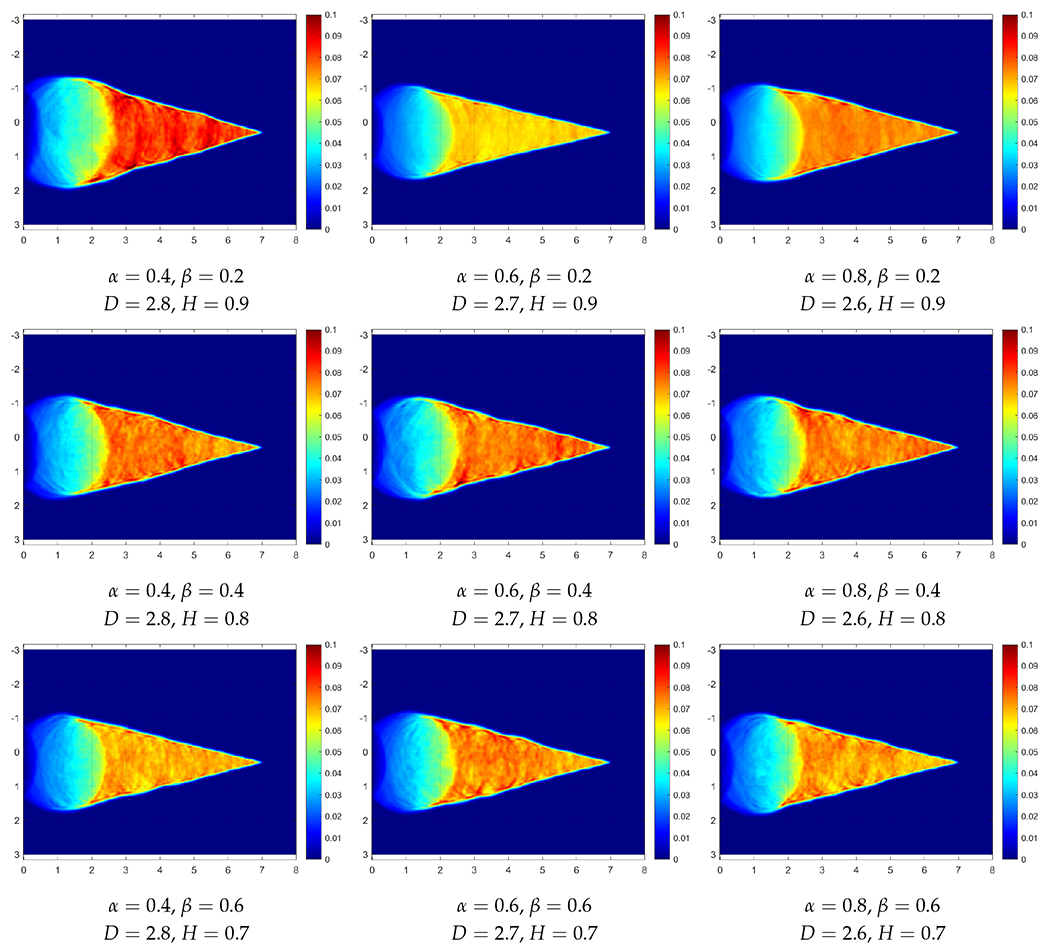

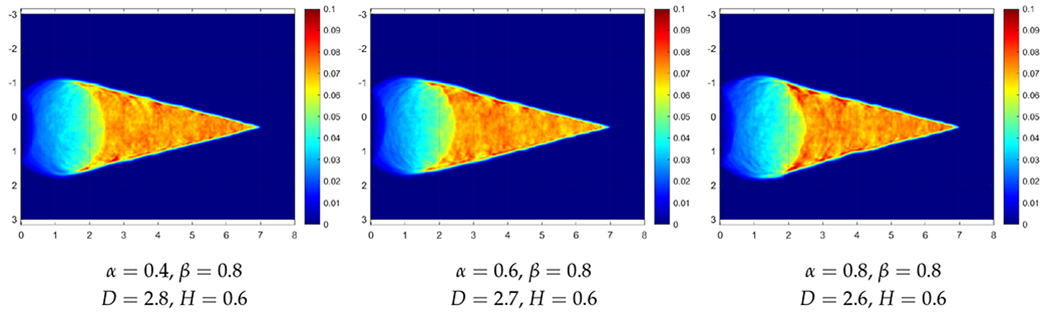
Simulation result at t = 3 in Dagum random fields with columns (from left to right) at *α* = 0.4, 0.6, 0.8 and horizontal rows (from top to bottom) *β* = 0.2, 0.4, 0.6, 0.8.

This section reports temperature fields obtained from solving sample realizations of RFs of the property *τ*. Simulations are performed in MATLAB with random fields generated by the RF package in R software. The panels of Figure 4 give such *T* fields for *τ* simulated as a Cauchy type RF for a range of *α* and *β* coefficients, with the mean 〈τ〉=4s and the variance *Var* = 0.4 s (recall Equation (11)). Similarly, the panels of Figure 5 give such *T* fields for *τ* simulated as a Dagum type RF for a range of *α* and *β* coefficients, also for 〈τ〉=4s and *Var* = 0.4 s (recall Equation (12)). In all the cases, the heat source *Q* travels on a straight path from the left to the right at a constant speed of *v* = 2 cm/s. Given that $c=\sqrt{\kappa /\tau }=0.5\;\text{cm}/\text{s}$, the nominal Mach number equals 4. This corresponds to a surgeon’s hand moving four times faster than the speed of second sound in a particular tissue.

The effects of material spatial randomness on the resulting Mach wedges are evident in all the panels and, as expected, they depend on the particular *α* (or *D*) and *β* (or *H*) values (recall (13)). To compare any particular temperature field (*r*) resulting from a realization of the random medium with the temperature field obtained on a homogeneous domain (*h*), a cross-correlation coefficient (or so-called normalized covariance) *ρ_rh_* is employed [17]:

(17) $$\rho_{rh}≔\frac{\frac{1}{I}\overset{I}{\underset{i=1}{\sum }}r(i)h(i)-\left[\frac{1}{I}\overset{I}{\underset{i=1}{\sum }}r(i)\right]\left[\frac{1}{I}\overset{I}{\underset{i=1}{\sum }}h(i)\right]}{\sqrt{\left\{\frac{1}{I}\overset{I}{\underset{i=1}{\sum }}r^{2}(i)-{\left[\frac{1}{I}\overset{I}{\underset{i=1}{\sum }}r(i)\right]}^{2}\right\}\left\{\frac{1}{I}\overset{I}{\underset{i=1}{\sum }}h^{2}(i)-{\left[\frac{1}{I}\overset{I}{\underset{i=1}{\sum }}h(i)\right]}^{2}\right\}}}.$$

This formula allows a quantitative cross-comparison of two identically sized panels. Thus, both fields, *r* and *h*, are obtained on rectangular domains of the same size: 438 × 250 = 109,500. The domain was cropped to remove the outer edge (dark blue region in the graphs) where nothing is happening. Thus, the *r* field is the set {r(i);i=1,…,I} while the *h* field is the set {h(i);i=1,…,I} where *I* = 109,500. The cross-correlation coefficients $\rho_{rh}$ so calculated are collected for various cases in Tables 1 and 2:

- Table 1 shows the cross-correlation coefficients comparing the results from Cauchy RFs with a homogeneous field of *τ* = 4 s.
- Table 2 shows the cross-correlation coefficients comparing the results from Dagum RFs with a homogeneous field of *τ* = 4 s.

The effects of changing values of *α* and *β* can be observed from Tables 1 and 2; the structure of these tables corresponds to the layout of Figures 2 and 3. For Cauchy RFs, smaller *α* and larger *β* values result in wedges that are more similar to the homogeneous case. On the other hand, for Dagum RFs, results from larger *α* and larger *β* are closer to the homogeneous case, except when *α* or *β* is equal to 0.8, where the correlations are high but the effect of changing the other parameter is not obvious from the cross-correlation coefficients.

Overall, even though the correlation numbers are not dramatically different from each other, the effects can actually be observed from the figures and simulation movies. To see the latter, the reader only needs to click on a select panel in Figures 4 and 5. From the Cauchy RFs in Figure 4, we can see that, as the fractal dimension *D* (which measures roughness) increases, many small waves generated by the material fluctuations end up smoothing each other out, causing the final result to be closer to the homogeneous case. On the other hand, as the Hurst coefficient *H* (describing the long-range memory) decreases below 0.5 and as the field gets more anti-persistent, we can see similar effects of smaller fluctuations causing a more homogeneous outcome.

Intuitively, we can see that the waves propagate more homogeneously on fields that are rougher locally on a smaller scale but have weaker fluctuations globally on a larger scale. For the Dagum RFs of Figure 5, which seem to be rougher overall than the Cauchy RFs, the effect of increasing the fractal dimension *D* is the opposite, which might indicate that the added roughness is not local enough.

## 6. Conclusions

The present study continues our investigations of mechanics problems on random fields with fractal and Hurst effects. Thus, except for some special cases, solutions in those prior studies as well as in the current problem of Mach wedges generated by supersonically moving heat sources have to be accomplished by Monte Carlo simulations of the resulting space–time fields of temperature. Overall, the sensitivity of wedges to the spatial randomness of the relaxation time (*τ*) is strongest in the case of random fields possessing the strongest fluctuations.

## Figures and Tables

**Table 1.** Cross-correlation coefficients, Cauchy random field.

	α = 0.2	α = 1.0	α = 1.8
*β* = 1.0	0.9983	0.9909	0.9843
*β* = 1.4	0.9923	0.9923	0.9922
*β* = 1.8	0.9992	0.9971	0.9956

**Table 2.** Cross-correlation coefficients, Dagum random field.

	α = 0.4	α = 0.4	α = 0.4
*β* = 0.2	0.8123	0.8827	0.9679
*β* = 0.2	0.9542	0.9569	0.9656
*β* = 0.2	0.9663	0.9739	0.9816
*β* = 0.2	0.9853	0.9840	0.9803

## Data Availability

Not applicable.
